# Disease activity level, remission and response in established rheumatoid arthritis: Performance of various criteria sets in an observational cohort, treated with anti-TNF agents

**DOI:** 10.1186/1471-2474-10-41

**Published:** 2009-04-23

**Authors:** Anders Gülfe, Daniel Aletaha, Tore Saxne, Pierre Geborek

**Affiliations:** 1 Dept of Rheumatology, Lund University Hospital, Lund, Sweden; 2 Medical University of Vienna, Vienna, Austria

## Abstract

**Background:**

Most composite indices of disease activity and response criteria in RA have been validated and compared in clinical trials rather than routine care. We therefore wanted to compare the performance of the DAS28, SDAI and CDAI activity indices, their activity states, their response criteria, and also compare with the ACR response criteria in an observational clinical setting.

**Methods:**

Agreement between the criteria sets was investigated using κ statistics in a non-randomized cohort of 1789 RA patients from southern Sweden, starting their first course of anti-TNF-treatment. Mean disease duration was 12 years. Completer analysis was used.

**Results:**

Agreement between high, moderate and low activity states was moderate or substantial, with κ = 0.5 or better for all criteria. Agreement between SDAI and CDAI disease states was > 90% in these categories with κ > 0.8. DAS28 original and modified cut point remission had good agreement (κ = 0.91). Agreement between responses was substantial at the overall/ACR20 level (about 95%, κ = 0.7 or better) for all criteria. By contrast, agreement was poor between moderate and high level responses.

**Conclusion:**

Disease activity states according to the various indices perform similarly and show substantial agreement at all levels except remission. Agreement between SDAI and CDAI states is excellent. Response criteria, applied at the individual patient level, are hard to interpret and show poor agreement, except at the lowest level of response. Thus, they should not be applied uncritically in clinical practice.

## Background

Indices of disease activity in RA, such as the Disease Activity Score in 28 Joints (DAS28) [1], the Simple Disease Activity Index (SDAI) [2] and the Clinical Disease Activity Index (CDAI) [3] and their respective cut-off levels for low disease activity (LDA) and remission (no activity) are tools that can be used in routine care. However, they have been validated mainly in clinical trials, where patients are meeting rigorous inclusion criteria and not always reflect the "real world" situation [4].

Response to treatment in rheumatoid arthritis (RA), as opposed to disease activity, denotes the improvement between two time points due to some intervention. In the trial setting, where the aim is to compare one treatment to another (standard) or none at all (placebo), response is the outcome measure of choice. The efficacy of a treatment is expressed as the proportion of a patient group that meets a certain response criterion. Indeed, disease activity indices, their LDA and remission criteria are not recommended as primary end points in trials due to low sensitivity to change[5]. On the other hand, response criteria, such as the DAS based EULAR moderate or good [6], SDAI [2] and CDAI [3] minor or major, and American College of Rheumatology (ACR) 20, 50 or 70% response [7], may be less suitable in routine care. Unlike the group level, individual responses will depend on the criteria set chosen, at least at the stricter levels [8].

The cut-points for the various activity indexes and the components of the response criteria are summarized in Table 1.

**Table 1:** Cut points for activity states according to various indexes (Panel A) and components of response criteria (Panel B)

***A. Activity index***								
	High	Moderate	Low	Remission				

DAS28 original#	> 5,1	< 5,1	< 3,2	< 2,6				
DAS28 modified cut off #	> 5,5	< 5,5	< 3,6	< 2,4				
SDAI##	> 26	< 26	< 11	< 3,3				
CDAI###	> 22	< 22	< 10	< 2,8				

***B. Response criteria***								

	TJC	SJC	Patient global VAS	Patient pain VAS	Evaluator global	HAQ	ESR	CRP

ACR	yes	yes	yes/no	yes/no	yes/no	yes/no	yes/no	yes/no
EULAR	yes	yes	yes	no	no	no	yes	no
SDAI	yes	yes	yes	no	yes	no	no	yes
CDAI	yes	yes	yes	no	yes	no	no	no

# DAS28 = 0,56x√TJC28+0,28x√SJC28x0,7xlnESR+0,014xPat global VAS(in mm)

## SDAI=SJC+TJC+Pat global VAS(in cm)+Eval global VAS(in cm)+CRP(in mg/dL)

### CDAI = SJC+TJC+Pat global VAS(in cm)+Eval global VAS(in cm)

Cut points for activity states according to various indexes (Panel A) and components of response criteria (Panel B). Yes, required; no, not required; yes/no, in the ACR criteria, 3 of the variables marked "yes/no" are required (0 or 1 laboratory measure). TJC, tender joint count; SJC, swollen joint count; VAS, visual analogue scale; HAQ, health assessment questionnaire.

The aim of the present study was to apply and compare the performance of various activity indices and response criteria in an observational cohort of patients from southern Sweden with established RA, treated with their first course of TNF-blockers. The findings may also serve as a reference of what levels of activity and response are to be expected in the "real life" setting without any formal or financial restrictions. The comparisons will also serve as a repository for future studies using one or the other measure as their primary end-point, relating them to results observable with other primaries.

## Methods

The South Swedish Arthritis Treatment Group (SSATG), a network of hospital and office based rheumatologists in southern Sweden, maintains a database into which all courses of treatment with biologic drugs for RA and other arthritides are entered as described elsewhere [9].

Patients eligible for the study had a diagnosis of RA, as judged by the treating rheumatologist, and started their first course of treatment with infliximab, etanercept, or adalimumab from March 1999 through December 2006. Due to the quality and safety monitoring character of the register, no formal ethics committee approval was required.

Patients were evaluated at baseline and 3 months. The follow up protocol included tender and swollen 28 joint counts, visual analogue scale (VAS) for pain and patient global, evaluator global Likert scale, ESR, and CRP. Thus DAS28, SDAI and CDAI could be calculated at each time point as well as fulfilment of EULAR, ACR, SDAI and CDAI response criteria at 3 months. The number of patients falling into the different categories of disease activity (including remission) when applying the cut off levels proposed for DAS28 (original and modified), SDAI and CDAI were then calculated.

### Statistical methods

Descriptive statistics was used throughout. For each level of disease activity and response, a reference criteria set was chosen. Patients fulfilling and not fulfilling this comprised the basis for comparison, as another criteria set was applied to the same patients. The agreement, i e patients fulfilling both sets (positive agreement) or neither (negative agreement) were then calculated as percentages and assessed with κ statistics[10]. The procedure was then repeated with a new reference criteria set. Thus, all comparisons were made between pairs of disease states and according to different indices, but each comparison was on the same level of disease activity. Likewise, pair wise comparisons of response criteria fulfilment at each level of response were performed. For example, DAS28 original cut point low was compared to SDAI low, and ACR20 was compared to EULAR overall response, etc. The κ values indicate the level of agreement beyond chance between two dichotomous variables. A frequently cited rule of thumb [11] is that κ values > 0.8 correspond to almost perfect agreement, 0.61–0.8 to substantial, 0.41–0.6 to moderate and 0.2–0.4 to fair agreement. κ = 0 denotes chance agreement. Completer analysis has been applied due to the observational nature of the study.

## Results

**Figure 1:**
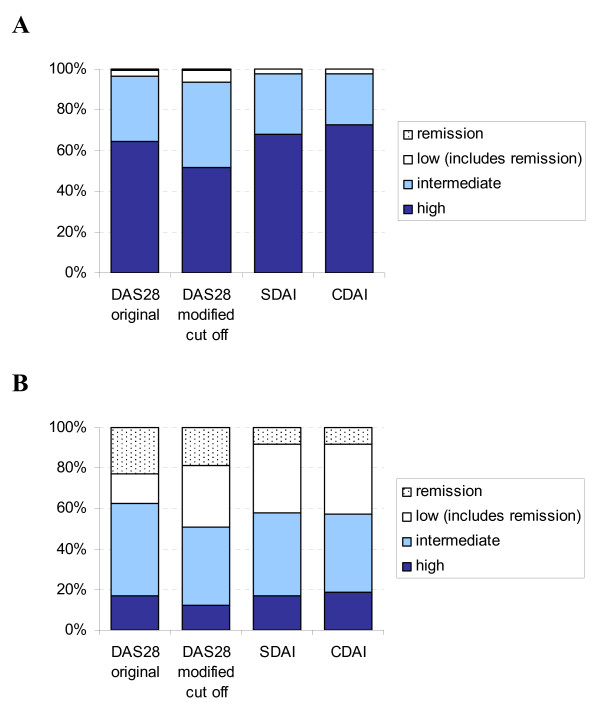
**Disease stages at A) baseline and B) at 3 month follow up according to the different criteria sets**. Notice that patients in remission are included in the low disease activity category, which thus comprises white and dotted areas together.

The eligibility criteria were met by 1789 patients. The baseline characteristics for all patients, the 1258 with 3 month data and the 531 patients lacking data at 3 months are given in Table 2. At baseline the majority (> 95%) of patients were in high/intermediate disease activity irrespective of criteria sets used (Figure 1A). At 3 months, 12–19% of patients had high, 39–46% had moderate, and 38–49% had low disease activity depending on criteria set. According to DAS28 original and modified cut points, 23 and 19%, respectively, were in remission, whereas about 8% were in remission according to SDAI and CDAI (Figure 1B).

**Table 2:** Baseline characteristics of all included and those with and without 3 month data

	All included N = 1789		With 3 month data N = 1258		Missing at 3 months N = 531	
	
	Mean	Std dev	Mean	Std dev	Mean	Std dev
Percent female	77.2		77.8		77.8	
Age	55.9	13.4	55.6	13.2	56.3	13.7
Disease duration, years	12.1	10.2	12.0	10.0	12.2	10.6
Ongoing DMARDs	0.85	0.57	0.85	0.57	0.84	0.57
DAS28 (0–10)	5.54	1.18	5.58	1.16	5.43	1.21
CDAI	30.6	12.3	31.0	12.1	32.2	13.9
HAQ (0–3)	1.34	0.64	1.36	0.64	1.29	0.62
CRP (mg/L)	30.8	33.0	31.4	33.6	28.9	31.3

The agreement between each defined disease state at baseline and 3 months, e.g. DAS28 low original cut point, DAS28 low modified cut point, SDAI low and CDAI low, are given in Table 3, along with their respective κ values. In general, agreement between the activity states determined by the cut-points of the various activity indices was moderate or substantial with κ values of about 0.5 or higher. At 3 months, remission according to the 2 DAS28-based categories had excellent agreement, κ = 0.84, and so did SDAI and CDAI, κ = 0.91, whereas agreement between DAS28-based agreement criteria and SDAI/CDAI was moderate, κ = 0.40–0.47. SDAI and CDAI had excellent agreement at all levels of disease activity (Table 3).

**Table 3:** Agreement between disease activity states at baseline and three month according to various indices, expressed as percentages and κ values.

**Baseline**							
***Valid N***		**DAS28 modified cut off**		**SDAI**		**CDAI**	
		***1657***		***1657***		***1681***	
	
**Reference criterion**	*Valid N*	**%**	**κ**	**%**	**κ**	**%**	**κ**

***High disease activity***							
DAS28 original	*1657*	52/35	**0,73**	60/27	**0,69**	61/24	**0,65**
DAS28 modified cut off	*1657*			50/31	**0,62**	51/27	**0,54**
SDAI	*1672*					67/26	**0,83**
***Moderate disease activity***							
DAS28 original	*1657*	29/55	**0,66**	23/61	**0,63**	20/62	**0,58**
DAS28 modified cut off	*1657*			25/52	**0,52**	21/53	**0,42**
SDAI	*1672*					24/69	**0,80**
***Low disease activity***							
DAS28 original	*1657*	2.7/94	**0,64**	1.3/96	**0,55**	1.3/96	**0,54**
DAS28 modified cut off	*1657*			1.8/94	**0,46**	1.6/94	**0,41**
SDAI	*1672*					1.7/97	**0,81**
***Remission***							
DAS28 original	*1657*	0.6/99	**0,91**	0.1/99	**0,15**	0.1/99	**0,15**
DAS28 modified cut off	*1657*			0.1/99	**0,18**	0.1/99	**0,18**
SDAI	*1672*					0/100	**1,00**

**3 month**							

***Valid N***		***1214***		***1224***		***1232***	
***High disease activity***							
DAS28 original	*1206*	12/83	**0,82**	13/79	**0,72**	13/78	**0.69**
DAS28 modified cut off	*1214*			11/82	**0,72**	11/80	**0.67**
SDAI	*1224*					15/80	**0,83**
***Moderate disease activity***							
DAS28 original	*1206*	34/50	**0,67**	32/46	**0,55**	30/45	**0,49**
DAS28 modified cut off	*1214*			29/50	**0,56**	26/49	**0,47**
SDAI	1224					35/55	**0,79**
***Low disease activity***							
DAS28 original	1206	38/51	**0,77**	33/53	**0,71**	32/52	**0,67**
DAS28 modified cut off	1214			39/47	**0,71**	37/46	**0,66**
SDAI	1224					40/55	**0,88**
***Remission***							
DAS28 original	1206	18/77	**0,87**	7.7/77	**0,42**	7.3/76	**0,40**
DAS28 modified cut off	1214			7.1/80	**0,47**	6.7/80	**0,44**
SDAI	1224					7.5/91	**0,91**

Percentages denote positive agreement/negative agreement, i.e. per cent patients achieving both compared activity states/per cent patients achieving neither.

**Figure 2:**
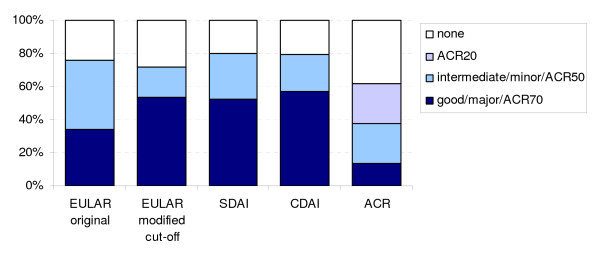
**Response at 3 months according to different criteria sets**. Notice that all ACR70 responders are include in the ACR50 responders, and all ACR50 responders are included in the ACR20 responders. Similarly EULARoverall responders include both good and intermediate responders, while SDAIoverall and CDAIoverall responders includes both major and minor responders

For the calculation of response at 3 months, 1258 patients had complete data, with baseline characteristics similar to the total cohort, but also to those with missing 3 month data (table 2). Response rates were 60–70% at the ACR20/overall level, 42% for EULAR original moderate, 19% for EULAR modified moderate, 25% for SDAI and CDAI minor response. At the major/good level, 35% responded according to EULAR original and ACR50 and 50–55% according to EULAR modified, SDAI and CDAI. Fourteen per cent were ACR70 responders (Figure 2).

The responder agreements at 3 months are summarized in Table 4. Substantial agreement (κ = 0.54–0.91) was found at the modest response level of ACR20, EULAR overall, SDAI overall and CDAI overall. Agreement at good/major level between EULAR original on one hand and, ACR70, SDAI, or CDAI on the other was poor (κ = 0.17–0.27), whereas EULAR modified was in much better agreement with SDAI and CDAI, κ = 0.69. SDAI and CDAI showed good agreement with κ values between 0.68 and 0.91 at the different response levels, while the agreement between other criteria sets at different response levels was more variable.

**Table 4:** Agreement of response according to various criteria sets at 3 months, expressed as percentages and κ values

***Reference criterion***									
		**EULAR**		**EULAR modified cut off**		**SDAI**		**CDAI**	
**Valid N**		**1163**		**1163**		**1184**		**1195**	
	
	**Valid N**	**%**	**κ**	**%**	**κ**	**%**	**κ**	**%**	**κ**
***Overall response***		***good+moderate***		***good+moderate***		***major+minor***		***major+minor***	
ACR20	1234	61/22	**0,61**	59/24	**0,62**	62/19	**0,54**	61/19	**0,54**
EULAR	1163			71/23	**0,83**	73/17	**0,71**	72/17	**0,68**
EULAR modified cut off	1163					70/18	**0,70**	69/18	**0,67**
SDAI	1184							78/19	**0,91**
***Moderate/minor response***		***moderate***		***moderate***		***minor***		***minor***	
ACR50	1234	13/33	**0,00**	3.5/47	**0,00**	7.3/41	**0,00**	5.6/45	**0,00**
EULAR	1163			14/54	**0,27**	13/43	**0,04**	10/46	**0,03**
EULAR modified cut off	1163					11/65	**0,31**	8.7/68	**0,28**
SDAI	1184							20/70	**0,74**
***Good/major response***		***good***		***good***		***major***		***major***	
ACR70	1234	12/64	**0,40**	13/46	**0,22**	12/46	**0,19**	12/42	**0,17**
EULAR	1163			31/43	**0,49**	25/38	**0,27**	26/36	**0,27**
EULAR modified cut off	1163					45/39	**0,69**	47/37	**0,69**
SDAI	1184							51/42	**0,86**

Percentages denote positive agreement/negative agreement, i.e. per cent patients fulfilling both compared response criteria/per cent patients fulfilling neither.

## Discussion

The major findings in this observational study of a non-randomized cohort of established RA patients, receiving their first course of anti-TNF treatment and followed for 3 months, were that the disease activity states according to the various indices performed similarly and showed moderate or substantial agreement at all levels except remission. SDAI and CDAI stages show excellent agreement. Agreement of response criteria is substantial at low response levels such as ACR20 and EULAR, SDAI and CDAI overall. At the moderate/minor level, when considered separately, agreement is poor, and this also holds true for the good/major level.

The κ value tells to which extent agreement is not explained by chance. The positive/negative agreements give, however, an idea of the degree of agreement; the closer total agreement (positive + negative) is to 100%, the higher degree of agreement. Furthermore, κ depends on sample size, and it is hard to interpret, if the compared groups are small. This is illustated by the limited κ agreement regarding both ACR70 responders and those reaching remission.

Completer analysis may lead to bias, if the drop-outs at 3 months substantially differ from the completers. The baseline data of the drop-outs and patients with 3 month data was therefore examined separately and not found to differ from the whole cohort in any clinically relevant way (Table 2). Also, in the observational setting, only patients remaining on therapy are contributing their data as time goes by, which tends to inflate the results. Missing information at 3 month follow up in our setting is higher than in the British Biologics register [12]. There may be several explanations for this, but the mandatory response demanded for drug continuation in the British setting may be one important factor. Also, the patients in the British register differ on numerous baseline characteristics such as higher disease activity and very high disability measured by the HAQ, all factors that may influence response rates and future disease states [12,13]. Thus each setting has its features and face to face comparison should always be done with great care.

The distribution between disease state categories at baseline and after 3 months is what may be expected in RA patients with mean disease duration of 12 years and who failed at least 2 different DMARDs. Achieving remission and even low disease activity thus appears not to be a too common event in established RA, treated with TNF-blockers. This is in agreement with other observational studies [16]. In our setting with many long-standing cases, erosive disease may preclude reaching remission in many patients although inflammation per se is suppressed by treatment. The small number of patients in low disease activity (LDA) at baseline is explained by patients taking high doses of prednisolone, also an accepted indication for commencing TNF-blocker treatment according to the Swedish guidelines.

The agreement between disease states, as defined by the DAS28 original and modified cut points, SDAI and CDAI proposed cut points, seems to be substantial. This is in agreement with comparisons based on trial data [14]. The excellent agreement of the disease state groups based on SDAI and CDAI irrespective of activity level may be accounted for by their great similarity: they contain the same components, added together, except for the CRP that is excluded from the CDAI. Our data thus support the notion of acute phase reactant values contributing little to the overall disease activity estimate [3]. The SDAI and CDAI remission is achieved in fewer patients than the DAS28 remission that appears less strict in this cohort, in agreement with previous findings [15]. At the remission level, agreement is almost perfect between EULAR original and modified cut off, and also between SDAI and CDAI at 3 months, as may be expected (Table 3). The moderate agreement between the DAS28-based and SDAI/CDAI remission, may partly be accounted for by the greater strictness of the latter (Figure 1B). Due to low patient numbers reaching remission, κ must be interpreted with caution in this category.

Response criteria are intended for clinical trials, and it is thus not surprising that they perform poorly when applied to individual RA patients in clinical practice. Agreement (Table 3) is variable across criteria and response levels with a tendency to be better at the less strict overall level, where EULAR moderate and good are merged to one group, as are SDAI and CDAI minor and major responders. In this manner, substantial agreement is achieved with ACR20, which includes all ACR50 and 70 responders, in accordance with previous findings [5,8].

The very poor κ values (often close to 0) for moderate response comparisons seem to indicate random agreement at the individual level. This may in part be due to the construction of the criteria. Thus, the ACR50 responders include all the ACR70 responders, whereas the EULAR moderate category does not include the EULAR good. The same mechanism may be operative concerning SDAI and CDAI minor and major responders. EULAR response according to original and modified definitions also exhibit poor agreement, especially at the moderate level. This is an expected finding, given the different cut-points of DAS28 used in each case. The value of response criteria in monitoring patients in routine care thus seems to be limited. In contrast, group level responses in a clinical setting can give some indication of the value of a particular treatment in routine care. As far as the management of individual RA patients with established disease is concerned, at least in our setting, the achievement of an absolute degree of disease activity seems to be a more relevant treatment goal than fulfilling a response criterion, i.e. achieving a given degree of improvement[8]. Our data thus do not support the use of response criteria as aid in the monitoring of RA-patients treated routinely with TNF-blockers, but this should be verified in other clinical cohorts.

In the development and evaluation of new treatment modalities, as well as in routine care, a unified concept of disease activity measurement and treatment aims will be beneficial. The widespread use of reproducible and simple composite measures of RA activity will facilitate this development. The present study provides support for this, but further validation of the indices in other cohorts is desirable.

## Conclusion

Disease activity states according to the various indices perform similarly and show substantial agreement at all levels except remission. Agreement between SDAI and CDAI states is excellent. Response criteria, applied at the individual patient level, are hard to interpret and show poor agreement, except at the lowest level of response. Thus, they should not be applied uncritically in clinical practice.

## Abbreviations

RA: rheumatoid arthritis; TNF: tumour necrosis factor; SSATG: South Swedish Arthritis Treatment Group; DAS: Disease activity score; ACR: American College of Rheumatology; SDAI: Simple disease activity index; CDAI: Clinical disease activity index; LDA: low disease activity; CRP: C-reactive protein; HAQ: Health Assessment Questionnaire.

## Competing interests

The authors declare that they have no competing interests.

## Authors' contributions

AG participated in the design of the study, performed the statistical calculations and wrote the manuscript. DA participated in the design of the study and suggested the statistical methodology. TS participated in the design of the study and helped drafting the manuscript. PG conceived the study and helped drafting the manuscript. All authors read and approved the final manuscript.

## Pre-publication history

The pre-publication history for this paper can be accessed here:


